# Bacteriuria in older adults triggers confusion in healthcare providers: A mindful pause to treat the worry

**DOI:** 10.1017/ash.2022.343

**Published:** 2023-01-09

**Authors:** Eva M. Amenta, Robin L.P. Jump, Barbara W. Trautner

**Affiliations:** 1 Center for Innovations in Quality, Effectiveness, and Safety (IQuESt), Michael E. DeBakey Veterans’ Affairs Medical Center, Houston, Texas; 2 Section of Infectious Diseases, Department of Medicine, Baylor College of Medicine, Houston, Texas; 3 Geriatric Research Education and Clinical Center (GRECC) at the VA Pittsburgh Healthcare System, Pittsburgh, Pennsylvania; 4 Division of Geriatric Medicine, Department of Medicine, School of Medicine, University of Pittsburgh, Pittsburgh, Pennsylvania; 5 Section of Health Services Research, Department of Medicine, Baylor College of Medicine, Houston, Texas

## Abstract

The evidence base for refraining from screening for or treating asymptomatic bacteriuria (ASB) in older adults is strong, but both practices remain prevalent. Clinical confusion over how to respond to a change from baseline, when to order a urinalysis and urine culture, and what to do with a positive urine culture fuels unnecessary antibiotic use for ASB. If the provider can take a mindful pause to apply evidenced-based assessment tools, the resulting increased clarity in how to manage the situation can reduce overtreatment of ASB.

Reducing the treatment of asymptomatic bacteriuria (ASB) is a priority for antimicrobial stewardship programs and the Infectious Diseases Society of America (IDSA).^1–3^ The treatment of ASB puts older adults in jeopardy of complications associated with antibiotic use, including adverse side effects and drug interactions, *Clostridioides difficile* infections, and the acquisition of antibiotic resistant organisms. Despite these risks, the rates of inappropriate use of antibiotics for ASB in nursing home residents remain high, ranging from 35% to 93%.^4^ A number of organizational guidelines and recommendations have highlighted the need to reduce treatment of ASB and to promote the use of simple decision aids to assist in diagnosis and empiric treatment of UTIs in older adults.^1–5^ However, a divide between recommendations and clinical practice remains.

## A confusing case

A scene unfolded in a long-term care facility: a nursing home resident appeared different than normal. When a caregiver talked to her more, the resident reported some irritation with urination. This situation triggered a concerned provider to order a urine culture. Two days later, the microbiology laboratory returned a positives urine culture result. The resident was feeling normal again and no longer complained of any problems related to urination. Upon learning of the urine culture results the following day, the provider ordered antibiotics.

**Fig. 1.**
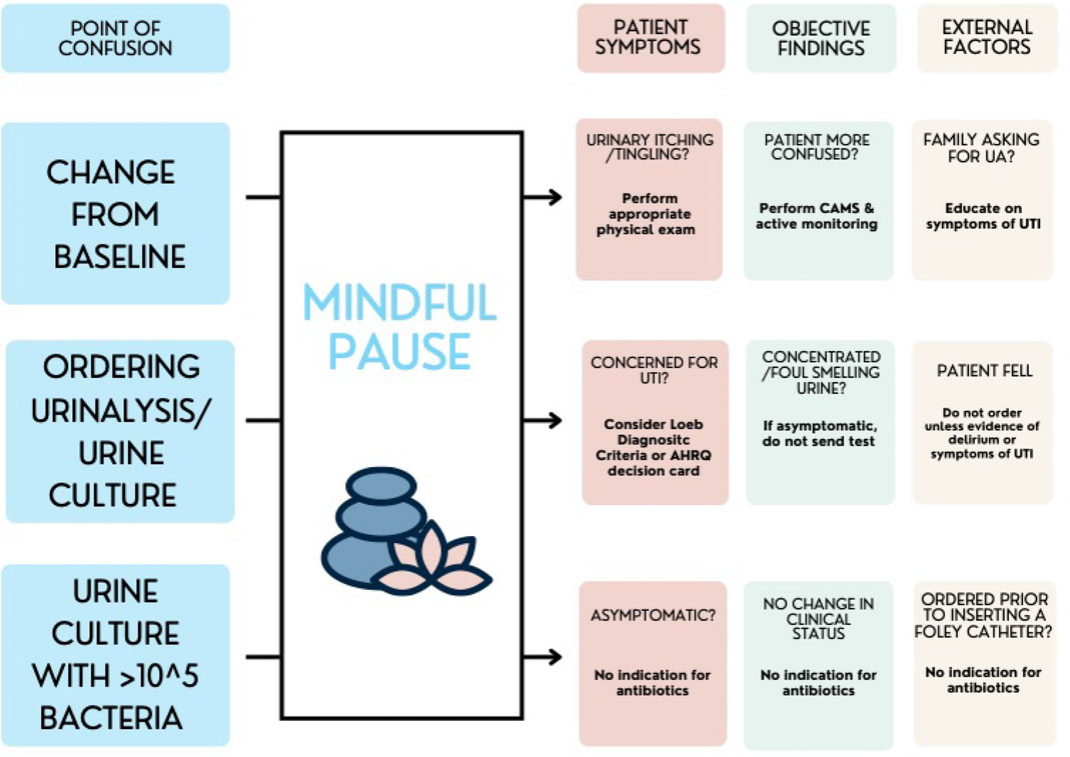
A mindful pause in diagnosis and treatments of UTI in long-term care residents. This decision aid can be employed by providers who are facing one of these clinical situations: a nursing home resident has a change from baseline, the provider is considering ordering a urinalysis or urine culture, or the provider receives a positive urine culture result. Each of these clinical situations can result in confusion. However, the provider should take a mindful pause and then follow the arrow to the recommended next steps for work-up and management in that clinical situation. These next steps are organized by patient symptoms, objective findings, and external factors.

This scenario had several potential intervention points. First, clinical concern surrounds changes from baseline status among nursing home residents. Second, a urinalysis and urine culture are often sent despite the absence of urinary symptoms in the resident. Third, positive urine cultures trigger antibiotic prescribing, often in the absence of re-evaluating the resident and even if the resident has since returned to baseline. A mindful pause at each of these intervention points can help providers redirect their efforts toward a more appropriate course of action (Fig. 1).

## A change from patient’s baseline

Nonspecific symptoms in older adults can create a challenging clinical picture, especially if the person seems confused or different than normal. Recognizing this challenge, the Society of Healthcare Epidemiology of America (SHEA) organized an expert panel to address the utility of nonlocalizing symptoms in nursing home residents. When a resident is more confused, the confusion assessment method (CAM) can be used to systematically identify delirium. If delirium is present, assessment for possible infectious and noninfectious etiologies of delirium should be performed. They note that delirium increases the risk of loss of functional status, hospitalization, and death.^6^ They specify that falls or behavioral changes other than delirium should not prompt work-up for infection. In addition, the updated McGeer criteria include delirium diagnosed by the CAM method as part of their constitutional criteria for infection.^7^ Using a standardized tool for diagnosing delirium can help identify delirium reliably and spur further action to identify the cause, which may or may not include infection.

Concerned family members may also notice a change in their loved one and request urine testing, particularly if they have been told that similar symptoms in the past were due to a UTI. A qualitative study performed by Lohfeld et al^8^ found that requests from family members were the reason providers at times felt compelled to order testing, and risk of incurring family dissatisfaction was reported as an obstacle to using a pathway to reduce unnecessary urine testing.

An additional point of confusion in determining whether an older adult does or does not have a UTI is that symptoms localizing to the genitourinary tract are not always indicative of a urinary tract infection. For example, burning with urination may herald a yeast infection (which can cause labial irritation), or dysuria may result from dehydration with concentration of the urine. Urinary obstruction or impaired voiding in men may be due to an enlarged prostate. Targeted questions are essential to clarify what an individual means when they report burning or difficulties with urination. If the initial course of antibiotics fails to resolve the person’s symptoms (assuming that the antibiotic given covers the organism isolated from the urine), additional antibiotics are unlikely to help. Instead, additional diagnostic investigations should be conducted to determine the real underlying cause of the patient’s departure from baseline.

A thoughtful, measured response to clinical changes is essential for evaluating older adults with communication challenges. Providers frequently default to ordering a urinalysis or urine culture because they are worried that they need to “do something” to address changes in a resident’s behavior or symptoms. Bacteriuria rarely merits antibiotic treatment (outside the scenarios of pregnancy and urologic procedures), and a mindful pause to consider nonurinary etiologies while observing the resident for evolution of symptoms can benefit both parties. The term “watchful waiting” does not fully capture what should be done and may not resonate well with the staff or family members caring for nursing home residents. A more apt description is “active monitoring,” which includes taking more frequent vital signs, encouraging hydration, and considering other reasons for the observed changes.^9,10^ Active monitoring allows time for the clinical picture to evolve and creates an opportunity to gather additional data. Once more information is available, interventions appropriate to address the underlying cause can be put into place. For example, relieving constipation will be more effective than antibiotics in a resident whose behavior change was prompted by constipation.

*Mindful pause 1: If there is a change from a resident’s baseline, take time to gather additional information, including serial vital signs, and use validated tools such as the CAM to assess delirium. Offer supportive care and consider a broad differential for the clinical changes*.

## A urinalysis or urine culture is considered

**Fig. 2.**
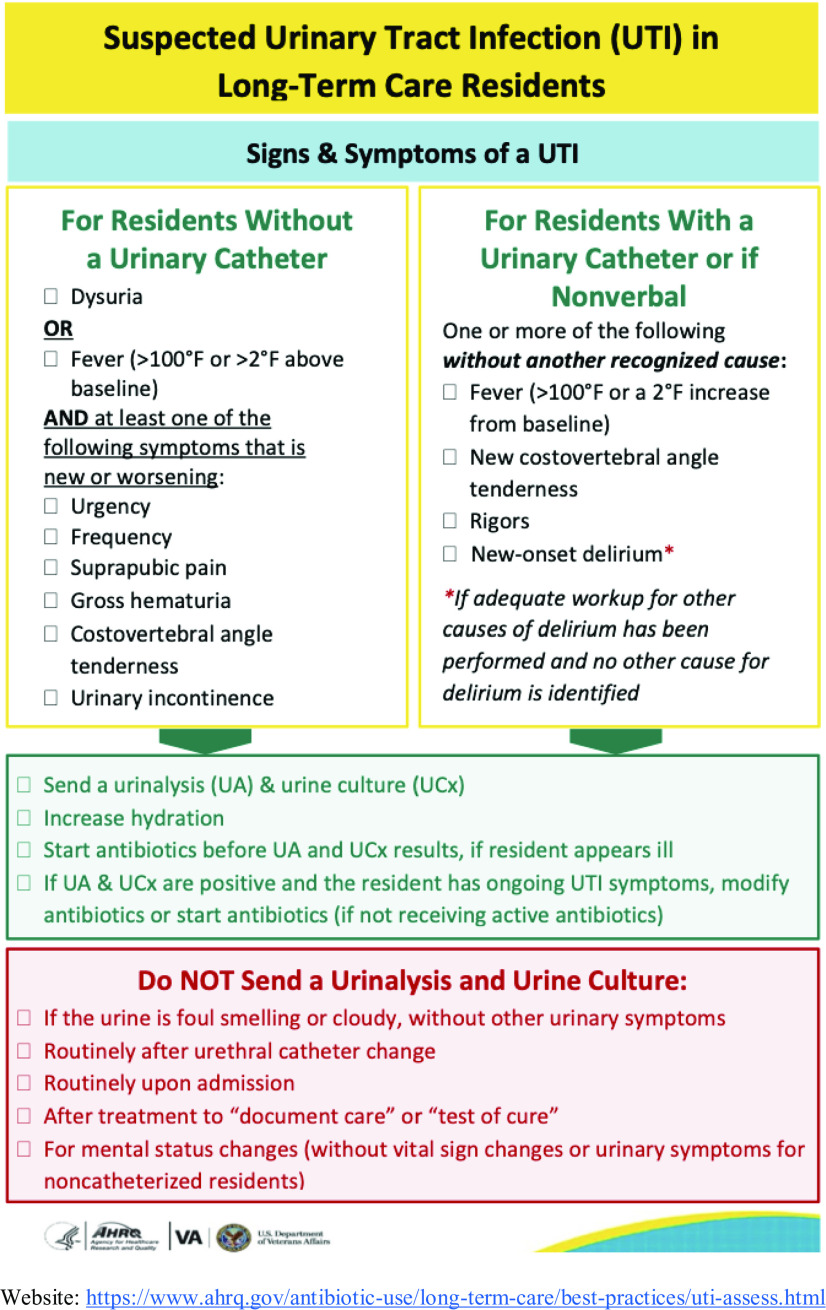
Agency for Healthcare Research and Quality (AHRQ) decision aid for suspected urinary tract infections in long-term care residents.

Frequently a provider receives an external request to send urine studies, from medical staff, family, or a colleague providing coverage. In such situations, validated clinical diagnostic tools may be useful in helping to determine the next step. The Loeb minimum criteria delineate subjective and objective findings that prompt initiation of antibiotics in long-term care residents for whom there is a concern for a UTI (Table 1).^11^ Using these criteria reduces orders for urine cultures from nursing home residents and does not lead to increased mortality or hospital admissions, which has been noted in a validation study among nursing home residents from 12 nursing homes in the United States and Canada.^11^ In addition, the Agency for Healthcare Research and Quality (AHRQ) has created a decision aid for when to suspect a UTI in long-term care residents for residents with and without catheters. These criteria support both diagnostic and antimicrobial stewardship (Fig. 2).^5^

**Table 1.** Loeb Minimum Criteria for Ordering Urine Cultures in Nursing Home Residents

If patient has a fever (>37.9°C) or 1.5°C increase above baseline on at least 2 occasions over the previous 12 hours **Plus** 1 or more of the following: dysuria, urinary catheter, urgency, flank pain, shaking chills, urinary incontinence, frequency, gross hematuria, and/or suprapubic pain
If patient has an indwelling urinary catheter **Plus** 1 or more of the following: new costovertebral tenderness, rigors, and/or new-onset delirium
If patient has new onset dysuria or 2 or more of the following: urgency, flank pain, shaking chills, urinary incontinence, frequency, gross hematuria, and/or suprapubic pain

Judicious ordering of urinalyses and urine cultures, commonly referred to as diagnostic stewardship, is an important component of antimicrobial stewardship programs. Diagnostic stewardship for urine testing is particularly important in nursing home populations because of the high prevalence of bacteriuria in residents. A urinalysis that is negative for both nitrites and leukocyte esterase essentially rules out acute cystitis. This type of result is uncommon among nursing home residents, however, because 25%–50% of female and 15%–35% of male residents have bacteriuria; of those 90% will have pyuria.^10,12,13^ A urinalysis from a nursing home resident that is positive for nitrites and leukocyte esterase has a specificity of ∼20% for diagnosing a UTI.^14^ To put these statistics in a clinical context, on the day a resident of a long-term care facility falls, if a urine culture is sent, there is a 50% chance that it will be positive. However, if the urine culture had been sent a week earlier, it would also have had a 50% chance of being positive. The fall and the concomitant bacteriuria are coincidental, not connected.

Antibiotic prescribing is the nearly inevitable response to a positive urine culture, even in the absence of symptoms.^15^ Diagnostic stewardship can help providers avoid ordering unnecessary tests, which in turn can reduce unnecessary antibiotic prescriptions. A focus on signs and symptoms that localize to the genitourinary tract should be emphasized over changes in the smell or appearance someone’s urine. Even the “cleanest” urine does not smell good. Observable changes in urine color or sediment frequently trigger concern, but such changes are nonspecific and even expected in older adults.^8^ Additionally, urine is readily accessible and thus easy to send for testing. Obtaining a good urine sample for microbiological culture, however, is not an easy process.^5^ Many people, let alone a frail or confused older adult, struggle to carry out the multistep process involved with obtaining a “clean catch” urine sample. For some nursing home residents, an in/out catheterization may be the only way to obtain urine suitable for microbiological culture—a potentially invasive, painful, and frightening process for some individuals.

*Mindful pause 2: Do not order a urinalysis or urine culture unless specific evidence-based criteria are met because rates of bacteriuria among older adults are high and the presence of bacteriuria is not synonymous with symptomatic infection*.

## Antibiotics are considered in response to a positive urine culture

When the urine culture results are available, and the report states that a quantity of a specific urinary pathogen above the threshold for UTI diagnosis, a provider who does not start antibiotics may feel that they are “withholding treatment.” Under those circumstances, avoiding an unnecessary antibiotic order requires mental strength and willingness to tolerate some cognitive uncertainty. The fear of missing bacteremia from a urinary source looms large. A study by Gharbi et al^16^ reported an association between either no prescribing or delayed prescribing of antibiotics for UTI diagnosis (diagnosed using *International Classification of Disease, Tenth Revision* codes) and subsequent *Escherichia coli* bacteremia in older adults in a primary care setting.^16^ This observation may raise concerns about active monitoring. However, limitations of study design, including the retrospective nature and unmeasured confounders significantly limit the applicability of their conclusions.^17^

Symptoms are key to the diagnosis and treatment of a UTI regardless of the results of a urine culture. Active monitoring gives the healthcare team an opportunity to observe whether the residents’ symptoms resolve or worsen to the point that they meet criteria for UTI testing and treatment, or whether another etiology emerges. Prescribing antibiotics is not a harmless action. Antibiotics contribute significantly to adverse drug events among nursing home individuals.^18^ Rates of *C. difficile* increase with the use of antibiotics, and the risk can last for weeks after completion of antibiotic.^19,20^ In addition, recurrence rates of bacteriuria are high after treatment with antibiotics, and the next round of bacteriuria is more likely to be caused by an organism resistant to the original course of antibiotics.^21^ Eventually the resistant organisms in the resident’s urine (and stool) may find their way into a normally sterile body cavity (bloodstream) or a wound, making subsequent infections more difficult to treat and leading to greater potential for harm.

*Mindful pause 3: Consider potential downstream harms of prescribing antibiotics in response to a positive urine culture. Does this patient have symptoms localizing to the genitourinary tract that are likely to be relieved by the antibiotics? If not, do not prescribe antibiotics in response to a positive urine culture*.

## Dissemination and uptake of best practices

Multifaceted interventions have successfully reduced ASB treatment in nursing homes. Nace et al^9^ randomized nursing homes to receive online courses, education cards, clinical vignettes, and virtual coaching calls. Nursing homes in the intervention arm had lower rates of antibiotics prescribed to residents unlikely to have acute cystitis and lower rates of *C. difficile* infections compared to nursing homes in the control arm.^9^ A virtual-learning collaborative initiative in nursing facilities in Ontario, Canada, found that nursing homes that participated in virtual learning sessions had lower rates of orders for urine cultures and antibiotic prescriptions per 1,000 resident days compared to those that did not participate.^22^ These findings are encouraging and suggest that implementing simple decision aids or guides in nursing homes may reduce inappropriate antibiotic use and associated adverse events.

For our resident who appeared different than normal, redirecting the provider’s initial impulse to order a urinalysis or urine culture into a mindful pause approach can help both her and her provider. The resident will avoid unnecessary antibiotics, and potentially harmful sequalae, while receiving a more thoughtful evaluation of the cause of her behavior changes. The provider will be more likely to correctly identify and treat the underlying etiology. Channeling provider actions into constructive activities, such as gathering more information (through validated tools such as the CAM), active monitoring, and applying diagnostic criteria (eg, the Loeb diagnostic criteria for UTI), gives everyone a pause to reconsider and re-evaluate. Such mindfulness then opens the differential to consider nonurinary causes of behavior change, and in turn helps resolve confusion among healthcare providers.
